# An ecological examination of teacher emotions in an EFL context

**DOI:** 10.3389/fpsyg.2023.1058046

**Published:** 2023-09-08

**Authors:** Yawen Han, Rining Wei, Jing Wang

**Affiliations:** ^1^School of Foreign Languages, Southeast University, Nanjing, Jiangsu, China; ^2^Department of Applied Linguistics, Xi’an Jiaotong-Liverpool University, Suzhou, Jiangsu, China; ^3^School of Foreign Languages, Nanjing Xiaozhuang University, Nanjing, Jiangsu, China

**Keywords:** teacher emotions, emotions, teachers’ professional development, EFL context, ecological system

## Abstract

Guided by a revised version of Cross and Hong’s framework based on Bronfenbrenner’s ecological system theory, the present study adopted a mixed-methods approach to explore teacher emotions in an EFL context. It aims to better understand teacher emotions and the influencing factors of these emotions in the microsystem, mesosystem, exosystem, and macrosystem of the above-mentioned framework. A total of 109 university EFL teachers completed an online questionnaire survey, and seven of them participated in the follow-up semi-structured interviews. The quantitative analysis of the questionnaire data revealed that the participants demonstrated different levels of the five focal emotions (joy, love, anger, fear, and sadness), and joy was the most frequently experienced emotion. Results also suggested that interactions with students, colleagues, leaders and family (from the microsystem), struggle with work-family balance (from the mesosystem), the performance evaluation system (from the exosystem) and the curriculum reform (from the macrosystem) emerged as important influencing factors of teacher emotions. Implications were discussed, and suggestions concerning emotion regulation were offered to EFL teachers working in tertiary institutions within China and similar contexts.

## 1. Introduction

In the past two decades, teacher emotions have attracted increased attention in the academic field (Chen and Cheng, 2022). Teacher emotions are important because they can significantly affect students’ learning outcomes and teachers’ well-being.^1^ However, there is a dearth of studies on teacher emotions utilizing an ecological perspective. Most extant studies tend to focus on one or two emotions and/or emphasize purely work-related factors that may affect teacher emotions.

Against this backdrop, the present study aimed to investigate a wide range of teacher emotions (including both positive and negative emotions) based on a revised version of Cross and Hong’s (2012) framework and explore both purely and non-purely work-related factors affecting teacher emotions. To this end, this study examined (1) the overall levels of joy, love, anger, fear, and sadness (viz. the five focal emotions) experienced by EFL teachers in Chinese universities, and (2) the factors, respectively, from the micro-, meso-, exo- and macro- systems that might affect these emotions when viewed from an ecological perspective.

## 2. Literature review

### 2.1. Research on teacher emotions

Teacher emotions are “socially constructed, personally enacted ways of being that emerge from conscious and/or unconscious judgments regarding perceived successes at attaining goals or maintaining standards or beliefs during transactions as part of social-historical contexts” (Schutz et al., 2006, p. 344). Teacher emotions are currently considered as a vital research focus because they can affect not only teachers’ own well-being (Gardner and Stough, 2002) but also students’ learning processes and outcomes (Yan et al., 2011; Frenzel et al., 2015).

One line of research on teacher emotions focusses on their influencing factors.^2^ Most studies along this research line tend to overemphasize purely work-related factors. For instance, Cross and Hong (2012) found that teachers experienced (1) great joy from their relationships with students, (2) sadness from the interactions with colleagues, (3) frustration and disappointment when the students’ parents could not help their children, as well as (4) anger and frustration in having to deal with the implications of the institution’s policies on instruction. A more recent study of Zhang and Tsang (2021) found that the institution’s performance evaluation system was a significant factor influencing teachers’ joy, sadness, frustration, anger, and fear, but not love of their profession. Compared with Zhang and Tsang (2021); Cross and Hong (2012) explored influencing factors of teacher emotions within an ecological system more comprehensively (see Section 3 for details).

Fortunately, a limited number of studies touch upon non-purely work-related factors that may affect teacher emotions (Chen, 2020). For instance, Chen (2016) utilized one item out of 26 items to discuss the influencing factor of work-family balance of teacher emotions in a self-developed questionnaire called Teacher Emotion Inventory (TEI). Other 25 items related teacher emotions to purely work-related factors such as teachers’ interactions with students, colleagues, superiors, and students’ parents. Utilizing a Turkish version of TEI, Atmaca et al. (2020) found that teachers from different subjects in Turkey experienced fear, which was linked to heavy workload and work-family imbalance. Accordingly, we suggest future studies need to give due attention to non-purely work-related factors (e.g., friends and family), which may help paint a full picture of the influencing factors for teacher emotions.

### 2.2. Research on language teacher emotions

Research into language teacher emotions has begun “gaining momentum only in the last decade” (Gkonou et al., 2020, p. 1), which was largely propelled by the research on teacher emotions reviewed in the previous section. Most studies targeing language teachers focus on one (e.g., Wang and Wang, 2015) or two teacher emotions (e.g., van Doorn et al., 2014). For example, focussing upon anxiety, Horwitz (1996) found that many non-native foreign language teachers experienced foreign language anxiety and that this anxiety might have negative consequences for language teaching for classroom instruction. More recently, Liu et al. (2022) again focussed on one singe emotion (anxiety) in the context of livestream language teaching among high school EFL teachers in China; from an ecological perspective, they found that anxiety was affected by teacher-student interactions, school authorities, students’ parents and technological support available for livestream teaching in the different layers of an ecosystem (see Section 3 for details). Although it was commendable that Liu et al. (2022) utilized an ecological perspective, they focussed only on one emotion. Covering a wider range of emotions within one single study offers richer information and generates more theoretical insight. This ultimately will benefit (1) comparison and contrast of empirical results concerning emotions and (2) accumulation of theoretical insight which will lay a solid foundation for theory development for research of emotions.

Similar to the above reviewed studies, Ergün and Dewaele (2021) also focussed on one single emotion (enjoyment) and found that about two thirds of the participants scored between four and five, indicating a high level of enjoyment (out of a scale of five, with a higher score indicating a higher enjoyment level) among a sample of 174 Italian as foreign language teachers. Although these authors aimed to examine potential influencing factors of enjoyment, they only focused on two variables (viz. resilience and well-being) and only included these focal variables in their regression analyses.^3^

In contrast, a very limited number of studies cover a wider range of teacher emotions in one single study, rather than simply investigating one or two emotions. For instance, in Cowie’s (2011) study, interviews with EFL teachers working in Tokyo universities revealed that teachers experienced joy, happiness, disappointment and anger in interactions with their students, colleagues and work. More recently, Yu et al. (2021) investigated the emotional experiences of 27 EFL teachers from Chinese universities in giving feedback on students’ writing, which turned out that teachers could experience positive emotions such as enjoyment and satisfaction as well as negative emotions such as disappointment and anger from giving students feedback. Put differently, Yu et al.’s (2021, p. 2) study covered at least four “discrete categories” of emotions in their study.

Given the benefits of focussing on a wide range of emotions (cf. Yu et al., 2021), the present study aimed to investigate five emotions (joy, love anger, fear, and sadness) from an ecological perspective with the following research questions (RQs):

*RQ1*. What are the overall levels of teacher emotions (e.g., love) for EFL teachers in Chinese universities?

*RQ2*. What factors influence these teacher emotions?

## 3. Conceptual framework

An ecological perspective helps to explain the influencing factors of emotions in teachers in general (Cross and Hong, 2012; Chen, 2020) and language teachers in particular (Liu et al., 2022). As Liu et al. (2022, p. 1) suggest, there is a dire need for more studies to examine language teachers’ emotions and other psychological individual differences (e.g., enjoyment, cf. Ergün and Dewaele, 2021) “by putting the teachers into the complex ecology.” Still there remains a lack of studies utilizing an ecological perspective. Our study endeavored to respond to this need by utilizing a revised version of Cross and Hong’s (2012) ecological system framework to explore a wide range of emotions among university EFL teachers and the influencing factors.

Cross and Hong’s (2012) ecological system, grounded in Bronfenbrenner’s (1979) theory, is believed to be “a useful framework for examining the immediate and distal environments in which teachers are embedded” (p. 958); this framework posits that individuals are constantly influencing and being influenced by the world around them; it helps to “tease out the layers of environment more systematically” (p. 959), which is useful in depicting a more comprehensive picture of factors affecting teacher emotions. The four layers (or levels) comprising an ecological system are: (1) microsystem that contains an individual’s roles and interpersonal relationships “in a given setting with particular physical and material characteristics” (Bronfenbrenner, 1979, p. 22); (2) mesosystem which describes “interrelations among two or more settings in which the developing person actively participates”(p. 25); (3) exosystem, viz. a number of settings that do not actively involve the individual but “affect, or are affected by, what happens in the setting containing the developing person” (p. 25); and (4) macrosystem, viz. the broader cultural context of an individual and influences interactions in all other layers. These systems are also influenced by the passing of time at the so-called chronosystem layer, which describes the influences of time both on these settings as well as on all interactions in the ecological system (Price and McCallum, 2015).

**Figure 1:**
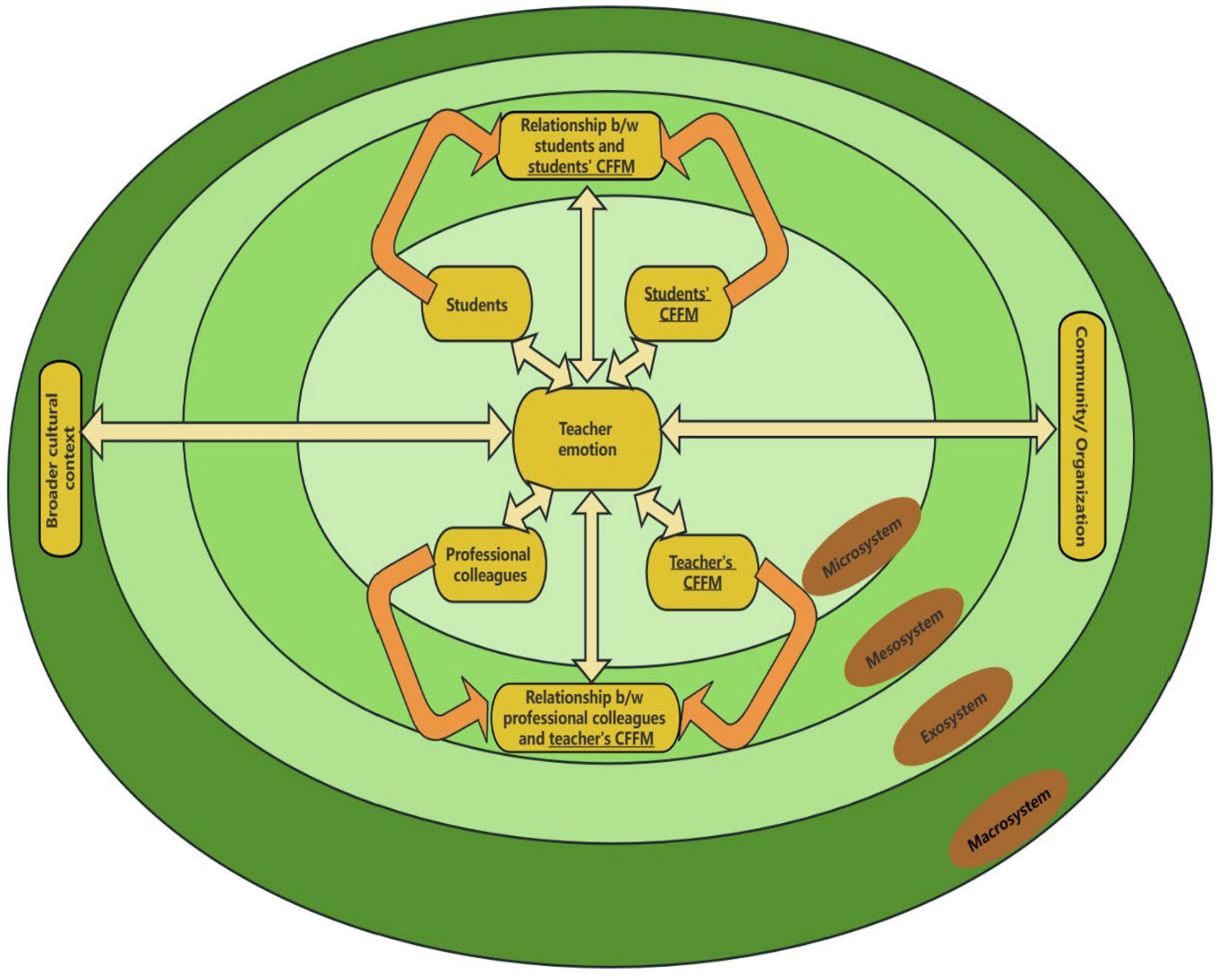
An ecological conceptual framework featuring stakeholders’ close friends and family members (CFFM) and other influencing factors of teacher emotion. The figure is adapted from Cross and Hong (2012) with permission from Elsevier.

Our revised framework differs from Cross and Hong’s (2012) in three major ways. First, in the original framework of Cross and Hong (2012), “administrators” and “colleagues” respectively constituted two micro-systems; now they are subsumed into one micro-system, labeled “professional colleagues.” Second, and perhaps more importantly, the micro-system of (students’) “parents” in Cross and Hong (2012) is expanded as “Students’ CFFM” because of Hofstadler et al.’s (2021) insight concerning the role of close friends and the fact that students’ guardians may not always be their parents (Wei, 2011). Third, based on both our experience and extant research (e.g., Chen, 2020), we suggest that it is instrumental to add a counterpart of the micro-system of “Students’ CFFM”: “Teachers’ CFFM” (see Figure 1 for details).

## 4. The study

### 4.1. Research context

China has the largest number of EFL learners/users in the world (Wei and Su, 2012; Wang and Wei, 2023). The best available data from government sources showed that the number of English learners/users in China already exceeded 390 million in 2000; although in the past two decades more updated data from government sources are not available, there is evidence suggesting that the number of English learners/users is on the rise (Wei and Su, 2015; Wei and Gao, 2022). Presumably the number of EFL teachers in China is astonishingly high.

Currently, the 3,000+ tertiary institutions in China can be categorized into three types: *yiben* (一本, first-tier), *erben* (二本, second-tier), and *sanben* (三本, third-tier) (Zhang and Wei, 2021). First-tier universities, including all of the Project 985/211^4^ universities, tend to focus on research. Both second-tier and third-tier institutions usually focus on either teaching or training students for future employment. Language teachers in first-tier universities, 985/211 universities in particular, are pressured to conduct and publish research, not to mention their teaching load. Performance evaluation systems in these universities are stringent. For example, one a first-tier Chinese university requires language teachers to publish a certain number of articles in leading international journals, in addition to satisfactory teaching evaluations, before they can be offered more stable employment contracts (Tao and Gao, 2021). In second-tier or third-tier universities, language teachers are also faced with pressure from the need to conduct research, which may not be as intense as the pressure faced by their counterparts in first-tier universities.

The recent curriculum reform of tertiary foreign language teaching in Chinese universities advocates the transformation from traditional English for General Purposes (EGP) to English for Specific Purposes (ESP) or English for Academic Purposes (EAP). This paradigm shift from EGP to ESP/EAP may be implemented from merely improving students’ English proficiency to cultivating their scientific research abilities. Focusing on students’ research abilities may facilitate the development of their abilities of critical thinking, communication, collaboration, creativity and cross-cultural competence, innovation, and scientific literacies (Cai, 2018, 2019). Moreover, in the past few years, the construction of “golden courses” (i.e., innovative and challenging high-quality courses) has been strongly encouraged by China’s Ministry of Education, which may add to the pressure of teachers. Constructing so-called “golden courses” has been a requirement for teachers’ academic promotion and career advancement against the backdrop of the on-going curriculum reform (cf. Yip et al., 2022).

EFL teachers in Chinese universities need to address the challenges ranging from teaching *per se* to providing administrative service (Tawafak et al., 2023), and to achieving continued professional development in the curriculum reform (Gao et al., 2018). They might feel insecure about their research ability and English proficiency. Indeed, EFL teachers in 985/211 universities face more challenges and higher pressure than their counterparts in non-985/211 universities. Focus on teacher emotions is essential, particularly when it comes to exploring teachers’ emotional reactions to the curriculum reform (Yip et al., 2022). As regards the reform of tertiary foreign language teaching in Chinese universities, studies have indicated the negative teacher emotions (e.g., anxiety) elicited by the reform (Wang and Wang, 2015; Yip et al., 2022).

### 4.2. Participants

Convenience sampling, which can “save time, money and effort” (Dörnyei, 2007, p. 129) in reaching participants, is used in this study. A total of 109 EFL teachers^5^ (13 male teachers, 96 female teachers) from different universities participate in this study. Among them, 12 participants are from 985 or 211 universities whereas the remaining 97 are from non-985/211 universities. Ten participants have Ph.D. degrees, whereas 86 and 13, respectively, have master’s and bachelor’s degrees as their highest educational qualifications. In terms of teachers’ developmental stage, there are 13 participants with less than 10 years of teaching experience, 39 participants with 10–15 years of teaching experience, and 57 participants more than 15 years of teaching experience. As regards academic ranks, 81 participants are lecturers and the other 28 are associate professors.

### 4.3. Instrument

A mixed-methods approach integrating quantitative and qualitative research, via questionnaires and semi-structured interviews, is adopted in the study. The triangulation of quantitative and qualitative data seems to “offer more justified insights” into exploration of teacher emotions (Xu, 2018, p. 45).

#### 4.3.1. Questionnaire

The questionnaire consists of two parts: the first part collects teachers’ socio-biographical information, including gender, educational background, years of teaching, academic rank and university type; the second part aims to understand teacher emotions in the form of the Likert 5-point scale. Each question has 5 options ranging from “never” (1 point) to “almost always” (5 points). The second part, adapted from Teacher Emotion Inventory (TEI)^6^ (Chen, 2016), encompasses items that could be “allocated into the four system levels (viz. microsystem, mesosystem, exosystem, and macrosystem)” (Chen, 2020, p. 5) from an ecological perspective. Considering the on-going curriculum reform in Chinese universities as mentioned above, we have adapted Chen’s (2016) TEI by removing some items more relevant to primary and secondary school teachers (e.g., “I feel pressured from high expectations of parents.”) and adding a few items more relevant to university teachers (e.g., “I feel disappointed when leaders expect too much of me in research.”).

The final version of the instrument includes 25 items (see Table 1 for details) along with five dimensions after excluding two items based on the results of Exploratory Factor Analysis (EFA).^7^ Reliability analysis of each dimension confirms a high level of reliability, with each Cronbach coefficient exceeding 0.80, indicating a high level of reliability. The reliability of the whole scale is highly satisfactory (Cronbach coefficient = 0.903). The factor loadings and other details are listed in Table 1. The final instrument comprises 15 items in the microsystem level, two items in the mesosystem level, three items in the exosystem level, five items in the macrosystem level, and no items in the chronosystem level.

**Table 1:** Teacher emotions at corresponding ecological system levels.

				Ecological system level			
	Factor loading	α	M (SD)	Micro-	Meso-	Exo-	Macro-
Love		0.90	3.97 (0.76)				
1. I love my teaching job because I could help my students adapt to future academic challenges.	0.87	0.87	3.92 (1.00)	√			
2. I love my teaching job because it is a profession which could obtain respect and recognition from society.	0.78		3.94 (1.01)				√
3. I am willing to carry out information teaching, reflective teaching, and language teaching	0.80		4.01 (0.80)			√	
4. I am willing to adapt to the curriculum reform.	0.76		4.04 (0.77)				√
Joy			4.39 (0.63)				
5. I am moved by my students’ sincere care.	0.82	0.89	4.45 (0.82)	√			
6. I am motivated by the support and care from leaders and colleagues.	0.79		4.08 (0.99)	√			
7. I enjoy sharing with my colleagues.	0.63		4.11 (0.85)	√			
8. I feel proud when I see my students make progress.	0.76		4.61 (0.69)	√			
9. I am moved for understanding and support from the society.	0.74		4.17 (0.99)				√
10. I am glad that my students enjoy my teaching.	0.69		4.66 (0.64)	√			
11. I am so excited when my students interact with my teaching.	0.73		4.65 (0.58)	√			
Sadness		0.82	4.08 (0.76)				
12. I am so sad with students’ negative feedback on my teaching though I’ve worked hard.	0.62		4.20 (1.06)	√			
13. I feel disappointed when my leaders ignore my efforts and contributions.	0.68		4.32 (0.83)	√			
14. I feel disappointed when I do not get what I should get.	0.53		4.05 (1.01)			√	
15. I feel disappointed when leaders expect too much of me in research.	0.73		4.17 (1.02)	√			
16. I feel disappointed when leaders expect too much of me in teaching (e.g. teaching competition, student context and postgraduate entrance examination rate).	0.59		3.65 (1.08)	√			
Anger		0.91	4.13 (0.90)				
17. I am indignant when the public blames teachers without any evidence.	0.83		4.16 (0.95)				√
18. I feel angry when I am treated unfairly in work arrangement and salaries.	0.70		4.10 (1.01)			√	
19. I feel angry when the society and public misunderstand our teachers.	0.80		4.14 (0.98)				√
Fear		0.84	3.75 (0.75)				
20. I am worried about how to improve student engagement and achievement.	0.63		3.73 (0.99)	√			
21. I am worried about competition with my colleagues.	0.65		3.15 (1.10)	√			
22. I am worried that students do not take responsibility for their study.	0.54		4.10 (0.96)	√			
23. I feel pressured from the imbalance of my work and life.	0.80		3.58 (1.13)		√		
24. I feel pressured when I suffer from shortage of time with too much work.	0.75		4.10 (0.92)		√		
25. I feel pressured when students cannot adjust to the changes in the teaching style in accordance with the new policy.	0.78		3.84 (0.88)	√			

#### 4.3.2. Semi-structured interviews

The benefit of using qualitative data from semi-structured interviews is that doing so helps complement the understanding based on quantitative data from the main questionnaire survey (Kong and Wei, 2019). The semi-structured interview questions were adapted from Chen (2020), who requested the interviewees to describe some emotion-related situations and two examples of an emotional context that impressed them most. We included the prompts from Chen (2020) for each interviewee with very minor modifications to the original wording. The initial version of the semi-structured interview questions was piloted with two university EFL teachers; based on their feedback, some minor stylistic adaptations were made before the interview questions were finalized.

### 4.4. Data collection and analysis

The research design received ethical clearance from the first author’s affiliation. Each participant, whose anonymity was assured of, provided written consent to take part in the present study. Electronic questionnaires were distributed via an online survey system called Wenjuanxing. They were sent to some university EFL teachers in China who showed willingness to participate in this study. These EFL teachers were then invited to forward the survey link to their colleagues via social media apps (e.g., WeChat) and email from June to July in 2020. We randomly contacted seven teachers (see Table 2 for interviewee information) out of the participants who indicated their willingness to join the follow-up online interviews by providing their email addresses at the end of the questionnaire. The Chinese language was used in both the survey and the interviews. The recordings were transcribed by the third author of the present paper; after the initial transcript was checked by the other authors for accuracy, some amendments were made before the transcript was finalized.

**Table 2:** Information of interviewees.

Teacher	Age	Gender	Teaching experience	Degree	Academic rank	Course(s) taught	Types of universities
T1	34	Female	8 years	PhD Candidate	Lecturer	College English	985/211
T2	34	Female	10 years	MA	Lecturer	English education	Non-985/211
T3	45	Female	22 years	MA	Lecturer	College English	Non-985/211
T4	55	Male	30 years	PhD	Associate professor	Linguistics	985/211
T5	41	Female	18 years	MA	Lecturer	Comprehensive English	Non-985/211
T6	39	Female	14 years	PhD	Associate professor	Academic English	985/211
T7	39	Male	12 years	MA	Lecturer	EAP	Non-985/211

RQ1 was addressed with descriptive statistics (e.g., mean), which was complemented with a series of follow-up *t*-tests conducted via SPSS 26.0. RQ2 was answered with both descriptive statistics (viz. percentages) generated from the quantitative data and content analysis of the qualitative data. Specifically, we coded and analyzed the interview data thematically and deductively following Elo and Kyngäs’ (2008) three stages: preparation, organizing and reporting; at each stage, guided by our conceptual framework, we took into account factors, respectively, from the micro-, meso-, exo- and macro- systems. Specifically speaking, each of us independently reviewed the finalized transcript and identified the patterns; then we gathered to examine findings and resolved disagreements after further discussion. In addition, we performed some type of member check (see Lincoln and Guba, 1985), a strategy to ensure data analysis credibility and trustworthiness.

## 5. Findings

### 5.1. Findings for RQ1: overall levels of teacher emotions

The participants demonstrated varying levels of the focal emotions (in descending order): joy (*M* = 4.39, *SD* = 0.63), anger (*M* = 4.13, *SD* = 0.90), sadness (*M* = 4.08, *SD* = 0.76), love (*M* = 3.97, *SD* = 0.76), and fear (*M* = 3.75, *SD* = 0.75). Furthermore, a series of follow-up one-sample-*t*-tests revealed that the means of joy, anger, sadness, love and fear were all statistically significantly (*p* < 0.0005) higher than the corresponding mid-point values (viz. 3), with the effect size^8^ (*r* = 0.91, 0.78, 0.82, 0.79, and 0.71 respectively), reaching “very large” benchmark.

To provide further information for future research to compare and contrast, we conducted a series of paired-sample *t*-tests to show the pair-wise comparison between the five focal emotions (see Table 3 for details). The comparison between joy and anger (Row 3 in Table 3) revealed a “very large” effect size (*r* = 0.26) with a very small *p* value (0.0003), suggesting that the level of joy was statistically significantly much higher than that of anger. In other words, joy had the highest level of presence amongst the five focal emotions.

**Table 3:** Paired samples *t*-test of the means of discrete teacher emotions.

	Mean difference	Std. error	Sig.	95% confidence interval for difference			
Teacher emotions				Lower	Upper	*t*	Effect size *r*
Joy-Love	0.42	0.06	<0.0005	0.29	0.54	6.41	0.52
Joy-Sadness	0.31	0.08	<0.0005	0.15	0.47	3.87	0.35
Joy-Anger	0.26	0.09	0.003	0.07	0.44	2.78	0.26
Joy-Fear	0.64	0.08	<0.0005	0.48	0.80	7.88	0.60
Fear-Love	−0.22	0.10	0.011	−0.42	−0.03	−2.32	0.22
Fear-Sadness	−0.33	0.07	<0.0005	−0.46	−0.20	−5.02	0.43
Fear-Anger	−0.38	0.075	<0.0005	−0.53	−0.23	−5.08	0.44
Sadness-Anger	−0.05	0.07	0.222	−0.19	0.08	−0.77	0.07
Sadness-Fear	0.33	0.07	<0.0005	0.20	0.46	5.02	0.43
Sadness-Love	0.10	0.10	0.145	−0.09	0.30	1.07	0.44

In summary, one concise answer to RQ1 is that the EFL teachers demonstrated different levels of the five focal emotions, with joy having the highest level of presence.

### 5.2. Findings for RQ2: factors affecting teacher emotions

Based on the conceptual framework (cf. Figure 1), the factors that influence teacher emotions were discussed in terms of the four different ecological system levels. The main findings were reported in respect to each of the levels as appropriate.

#### 5.2.1. Microsystem: interactions with students, colleagues, leaders and family

##### 5.2.1.1. Interactions with students

According to the survey and semi-structured interviews, teachers had positive emotions with students, which was in line with Chen (2020). Teachers experienced love and joy when they could facilitate students’ future development and progress or gain students’ recognition and affirmation. 76.1% of the teachers loved teaching because they could help students adapt to future academic challenges. 94.5% of the teachers were glad that the students enjoyed their teaching. 92.6% of them were proud of what students had achieved. 89.9% of the teachers were very moved by student care, which could be illustrated in the following interview excerpt.

Extract 1

“I tutored a student (a non-English major) for an English competition after class and she won a good prize. Her interest in foreign languages has maintained, and she even considered a career related to foreign languages. After she graduated, she would send me messages on important festivals such as Teachers’ Day or Spring Festival. It has been many years. I am very touched.” (T3)

Up to 78% of the teachers stated that they feared that students were not responsible for their own learning. A similar percentage (71.5%) feared because students could not adjust to the new teaching style. Over 80% (81.7%) of them felt sad because they tried their best to teach students but got negative feedback on the teaching. T5 expressed these emotions (as indicated in Extract 2).

Extract 2

“I assigned homework, asked students to recite English speeches, and required students to prepare for the reading task before class. Some students thought it was too much and showed their dissatisfaction in unpleasant remarks via QQ platform, which happened to be read by me. I was so sad because I hoped that they could improve language skills in this way, but they thought it was simply a burden.” (T5)

T4 also expressed anxiety he experienced when teaching in a language that was not his native language. This was in accordance with Horwitz’s (1996) finding that EFL teachers also suffered from language anxiety because they taught in a language that was not their native language, which may bring them tremendous anxiety and uneasiness Extract 3 lent support to this finding.

Extract 3

“I have never been abroad, and I don’t have a good command of English. Every class is a challenge. This challenge and anxiety continue to exist unless I retire and have no classes.” (T4)

##### 5.2.1.2. Interactions with colleagues

As high as 78% of the teachers reported that they were motivated because of their colleagues’ support. T2 also showed enjoyment in cooperating with colleagues.

Extract 4

“Teams are required in teaching competitions. Applications for scientific projects also require collaborators. Collaborators can save time and effort. They have multiple perspectives. Cooperation makes tasks easier and the whole process more enjoyable.” (T2)

This confirmed that the teachers’ relationships with their colleagues were often a source of satisfaction, especially when there was emotional warmth based on friendship, respect, and collegiality (Cowie, 2011; Karagianni and Papaefthymiou-Lytra, 2018).

However, negative emotions (e.g., pressure) could also be found, which might stem from the envy of colleagues with strong academic research capabilities (see Extract 5) or inclination to maintain a good relationship with colleagues who show little interest in conducting research (see Extract 6).

Extract 5

“Some colleagues did well in scientific research, so I was also very envious. In fact, I wondered why they could excel in scientific research. Of course, I was definitely under pressure. However, I am not ashamed of being a lecturer. An experienced teacher in our university is a lecturer.” (T5)

Extract 6

“To get along well with my colleagues who do not engage in scientific research, I do not want to be actively engaged in scientific research. However, without publications, I feel sad.”

These findings echoed previous research finding that university foreign language teachers produced negative emotions when they got along with their colleagues (Cowie, 2011).

##### 5.2.1.3. Interactions with leaders

According to the survey results, 78% of the teachers were motivated by the support of leaders, who most university EFL teachers contact directly are deans of the university. Extract 7 lent support to this finding.

Extract 7

“Leaders encourage us to participate actively in various academic conferences, support us in terms of funding, invite experts to give lectures and organize some academic discussions. We are very encouraged.” (T2)

Emotions of sadness and fear were related to the expectations and attitudes of the leader. Up to 83.5% of the teachers felt frustrated because their leaders ignored their hard work and efforts; 80.7% of them were under great pressure because of the high expectations from their leaders for scientific research, which could be supported by one emotion-related situation of T3.

Extract 8

“When leaders see us, they always ask how we are conducting the scientific research recently. I always say that I am very busy. In fact, we have a lot of work besides scientific research, but the leaders are more concerned about the results, and we feel pressured sometimes.” (T3)

The influence of leaders on teachers could be reflected in their relationship prescribed by the hierarchical order in the Chinese culture that the one of a lower status (teachers) ought to listen to the one of a higher position (leaders) (Xu, 2013).

##### 5.2.1.4. Interactions with family

The following extracts showed that the support and understanding of family, although not covered in the questionnaire, had a positive influence on teacher emotions.

Extract 9

“My parents helped me take care of my children and take the housework, which saved a lot of time for me and allowed me to do more things I wanted to do. I am very grateful and feel very happy.” (T1)

Extract 10

“When I wrote my research project, my husband could help me with his AI technology. This indeed saved me a lot of time and trouble.” (T6)

Without support and understanding of family members, teachers might experience negative emotions such as frustration, loneliness as well as sadness, which could be shown in Extracts 11 and 12.

Extract 11

“I did not devote much to taking care of children or housework. My family members would question the reason why I devoted so much to work and wondered what outcomes I could get from so many efforts. Lack of their understanding, I felt frustrated and lonely.” (T2)

Extract 12

“My wife likes to nag about trivial matters. I have to listen to her and respond or she will be furious. I can’t concentrate on work at home. Sometimes I felt sad, but I could do nothing about it.” (T4)

#### 5.2.2. Mesosystem: struggle with work-family balance

The balance between work and family emerged as an important factor in the mesosystem, as reflected by both quantitative and qualitative findings. On the one hand, over half (57.8%) of the teachers felt pressured from the imbalance of work and family. On the other hand, the interviewees expressed negative emotions (e.g., sadness and helplessness), which echoed Atmaca et al.’s (2020) finding that work-family imbalance caused negative emotions. The follow excerpts illustate some negative emotions arising from the struggle of some female teachers when it comes to striking a balance between work (e.g., the need to conduct scientific research) and family obligations (e.g., having to take care of the young children).

Extract 13

“We have to consider family. Many things engage me and now the energy and time for scientific research are actually very limited, so it is difficult to publish high-quality articles in a short period of time. Sometimes I feel anxious and helpless as I have to spend most of my time caring for my two-year-old son.” (T2).

Even for more senior teachers with older children, things did not seem easier because they had to care for both the aging parents and their children. In China, children and most college students (aged 18 or more) depend on parents for their needs (Taormina, 2017), not to mention very young children. With a child under seven in age, T7 shared his dilemma where he had no extra energy to do scientific research, although he needed research publications in top-tiered international journals to apply for academic promotion:

Extract 14

“My parents, both of whom have retired, are not in good health and need to be taken care of. Their health conditions prevent them from helping the caring of my daughter who will go to Primary School in the upcoming semester. My wife and I shoulder the main responsibility of caring for three people in the family. I have no extra energy to engage in scientific research. My way to promotion is tough, so I feel sad because my leader tells me that I need two more SSCI-indexed papers before I can be promoted to Associate Professor.” (T7)

#### 5.2.3. Exosystem: the performance evaluation system

According to the survey, teacher emotions such as anger or disappointment stemmed from the performance evaluation system in the exosystem. A large faction (80.8%) of the teachers felt angry because of unfair treatment in salaries; 74.3% of them felt disappointed because that they did not get what they should get.

According to the interviews, the performance evaluation system, which imposes the requirement of publications in core journals in particular, possibly triggers negative emotions like worry, anger or disappointment as shown in Extract 15 and 16; this finding echoed with that from Zhang and Tsang (2021). Teachers experienced these emotions possibly because the performance evaluation system disempowered them to control their work and subjected them to external monitoring regarding the heavy workload that they cannot handle. Many teachers were worried and felt that it was difficult to get published in core journals. On the one hand, they might feel that they were not capable of carrying out scientific research (Liu, 2022). On the other hand, they might lose confidence in attempting to publish in core journals because of too many failures. However, in most universities, without research outputs, the way to academic promotion is difficult (Tao and Gao, 2021), which could produce negative emotions in teachers.

Extract 15

“The university scientific research policy has been changing. Previously, universities awarded teachers when they published articles in provincial-level academic journals. Now if we have no publications in core journals and cannot meet the requirement for research outputs, there will be punishment (e.g. deduction in the annual perks). This makes me worried.” (T2)

Extract 16

“I really want to retire early. Now that the requirements for scientific research are so high, I don’t know if my academic rank will be downgraded if I don’t publish in core journals. I am angry and disappointed by the high requirements. I wish I had transferred to higher vocational institutions when I had the opportunity.” (T3)

Besides the performance evaluation system, teachers were also worried about the instability of core curriculum systems and policies in universities, as can be indicated in Extract 17.

Extract 17

“In the teaching reform, we are anxious and nervous. It is to break up all the modules of our current courses, and then we have to change the teaching materials, but in fact there is no need at all because it is just a minor change. The textbook, teaching mode and teaching methods have not changed, so I don’t know what the purpose of the reform is.” (T1)

#### 5.2.4. Macrosystem: the curriculum reform

The quantitative results showed that a large proportion (78.9%) of the teachers indicated willingness to adapt to the curriculum reform. The qualitative results (see Extract 18, 19, and 20 for details) also showed that teachers felt pressured to adapt to the reform in response to the transformation from traditional EGP to ESP or EAP and the advocate for the “golden courses” standard. They worried about their competence in teaching ESP/EAP.

Extract 18

“In response to the curriculum reform, my team leader assigned me to teach medical English in the next semester. The leader told me to teach EAP to a group of medical students, even though I never taught EAP courses and knew nothing about medical science. I am very helpless because I am not well trained to do this teaching assignment. I have tried my best to read some textbooks on EAP and watch video-recorded demonstration classes. Whenever I think of this challenging assignment at night, I feel anxious and have difficulty falling into sleep.” (T1)

Extract 19

“I don’t have research and publication experience. How can I convince my students of my ability? In this information society, it is easier for students to get knowledge. In class, I am not always sure about my teaching. I worry that the teaching doesn’t meet the requirement of ‘golden courses’. I am anxious.” (T7)

Extract 20

“Last semester I taught an EAP course for engineering students. In terms of vocabulary teaching, I used a word list, which I had assumed to be useful for the development of their academic English proficiency. However, I received some complaints from students that the word list was too general, which was not helpful for their academic exchanges in their discipline (e.g. doing oral presentations at research seminars and conferences). This feedback disheartened me.” (T6)

Our findings echoed Yip et al.’s (2022) finding that teachers tended to experience highly negative emotions and were susceptible to vulnerability when they felt the risks and threats that the curriculum reform posed to them.

To sum up, a concise answer to RQ 2 is that (1) in the microsystem, teachers’ interactions with different stakeholders (viz. students, colleagues, leaders and family) influenced their emotions, (2) in the mesosystem, struggle with work-family balance was an important influencing factor, and (3) the performance evaluation system and the on-going curriculum reform in the exosystem and the macrosystem also influenced emotions.

## 6. Discussion

This study examined the overall levels of teacher emotions for EFL teachers in Chinese universities. One important finding was that among the five emotions, joy was the most frequently experienced emotion related to different stakeholders (e.g. students and colleagues); this was in line with previous studies conducted in different parts of the world including China (Chen, 2020), Italy (Ergün and Dewaele, 2021), and Turkey (Atmaca et al., 2020). The high level of joy (*M* = 4.39 out of 5) found in our study may be attributed to two factors. First, teachers are more valued by Chinese society in terms of social status compared to their counterparts in many western countries (Atmaca et al., 2020). Second, half of the participants have more than 15 years of teaching experience, and these *experienced* teachers may be more skillful in interacting with different stakeholders and more adapted to the job requirements. Accordingly, they may have a high level of joy during teaching. Our result resonated with Chen’s (2020) finding that length of teaching experience was an important factor affecting teacher emotions.

Another important finding from our study was that work-family balance was identified as an important factor affecting teacher emotions. This result was hardly surprising in the context of Chinese culture, which is collective and familial (Hofstede and Bond, 1988). As such, teachers in China tend to believe that family interests are higher than work (Hong et al., 2021). This belief has its roots in Confucianism, one key component of which emphasizes the obligations of family members to the family (Yang, 2004); this has exerted significant influence on the thinking and behavior of every Chinese (Xiong and Wei, 2020). Understandably, the teachers in our study (such as T7) tend to prioritize their family responsibilities over work when having to choose between work and family obligations. That said, our finding of prioritizing family obligations over work requires corroboration and verification from future studies conducted in contexts that are less affected by Confucianism.

Our findings concerning negative emotions, which were produced in interactions with colleagues, were consistent with the previous findings (Cowie, 2011; Chen, 2020). The negative emotions were triggered by a dilemma EFL teachers faced in dealing with collegial relationship. On the one hand, in the Chinese society where collectivism is usually valued over individualism in the workplace, teachers endeavoring to conduct research are expected to maintain a harmonious relationship with their colleagues who are not interested in conducting research. Maintaining such a harmonious relationship may hinder teachers’ endeavor for conducting research. On the other hand, the performance evaluation system, which determines teachers’ academic promotion and career advancement, requires them to focus on individual excellence (e.g., producing research publications in core journals). To achieve academic promotion and/or career advancement, teachers should attempt to excel in the required aspects according to the performance evaluation system. This dilemma might put teachers in a negative collegial relationship, thus producing negative emotions (e.g., sadness).

## 7. Conclusion

The findings have several theoretical and practical implications. Theoretically, the study confirmed the applicability of the revised version of conceptual framework in exploring factors of teacher emotions in an EFL context. The findings have confirmed the value of adding a layer of factors: close friends and family members (CFFM). Specifically, the importance of including family, a non-purely work-related factor, in teachers’ ecology has been illustrated in the following findings: (1) teachers’ interactions with family comprised an important component in the microsystem; and (2) the balance between work and family represented a crucial factor in the mesosystem. All in all, the present study has demonstrated the usefulness of the proposed framework (see Figure 1) in examining EFL teacher emotions. The proposed framework can be utilized in future studies and is open to potential validation and/or modification by new empirical data from further research endeavors.

In addition to the above theoretical insights, the study also has practical implications. Different stakeholders should exert joint efforts to improve teachers’ emotional well-being and ultimately their teaching. For example, teacher educators might like to consider offering training courses, which help teachers to fully understand that (1) the factors affecting their emotions can come from *both* the work *and* non-work environments, and (2) emotions can be regulated. These training courses may focus on the best practices of emotion regulation. During the training, teachers can be given different scenarios to practice both preventative and responsive strategies of emotional regulation; the preventative methods include thinking about the positive aspects of teaching whereas the responsive methods comprise self-talk, deep breathing, using emotional intelligence tactics and other strategies (Sutton, 2004; Jiang et al., 2016; Smith and King, 2018; Al-Obaydi et al., 2022). Furthermore, teachers’ family members may reflect on what can be done to mitigate negative teacher emotions and improve positive teacher emotions; for instance, mini-workshops can be held for teachers’ families to exchange good practices of supporting the teacher family members toward better emotional well-being outside the work environment.

As mentioned above, the on-going curriculum reform in China requires many EGP teachers to switch to EAP/ESP, and this challenging shift led to negative emotions in some participants, which was detrimental to their emotional well-being. One implication is that teacher educators and policymakers should provide sufficient training for teachers to reduce their uncertainties regarding EAP/ESP teaching, so as to alleviate teachers’ emotional burden and enhance their teaching efficiency. Without sufficient professional support (e.g., The Collaborative Electronic Diary Exchange Project; Karagianni and Papaefthymiou-Lytra, 2014), it would be impossible to build up a strong and emotionally-healthy workforce in EFL. These suggestions should be applicable in many tertiary institutions within China and possibly beyond.

However, two limitations of this study should be acknowledged. First, the participants in this study were based on convenience sampling. Thus, the findings reported here cannot be generalized to all EFL teachers. Second, due to the relatively short time frame of the study, the findings may not reflect the dynamic picture of EFL teachers’ emotional change. Moreover, the chrono-system of EFL teachers’ ecology remains unexplored. To address these limitations, two future lines of research are worthy of pursuing: (1) involving more teachers from different contexts; and (2) conducting longitudinal research so as to track teachers’ emotional fluctuation. Specifically, for example, in connection with the first research line, it would be useful for future studies to follow the following steps: (1) investigating EFL teachers in non-tertiary in the Chinese EFL context; (2) going beyond the Chinese EFL context, it is useful to focus on teachers in other EFL contexts such as Japan, South Korea, and Thailand to see if a different set of results might emerge; and (3) accumulating sufficient empirical data to pave the way for the exploration of mechanisms.

## Data availability statement

The original contributions presented in the study are included in the article/supplementary material, further inquiries can be directed to the corresponding authors.

## Ethics statement

The studies involving human participants were reviewed and approved by School of Foreign Languages, Southeast University. The patients/participants provided their written informed consent to participate in this study.

## Author contributions

YH: conceptualization, funding, in-depth data analysis, original draft preparation, and supervision. RW: data collection, preliminary data analysis, and original draft preparation. JW: conceptualization, in-depth data analysis, original draft preparation, reviewing, and editing. All authors listed have made a substantial, direct, intellectual contribution to this original research, approved it for publication.

## Conflict of interest

The authors declare that the research was conducted in the absence of any commercial or financial relationships that could be construed as a potential conflict of interest.

## Publisher’s note

All claims expressed in this article are solely those of the authors and do not necessarily represent those of their affiliated organizations, or those of the publisher, the editors and the reviewers. Any product that may be evaluated in this article, or claim that may be made by its manufacturer, is not guaranteed or endorsed by the publisher.

## Footnotes

^1^Researchers have extensively explored the important outcomes vis-a-vis teacher emotions, such as classroom management, students’ academic achievement, professional development and teacher burnout (Gardner and Stough, 2002; Valiente et al., 2012; Hagenauer et al., 2016; Hansen and Mendzheritskaya, 2017; Atmaca et al., 2020; Chen, 2020).

^2^When teachers are regarded as “part of an education system which includes policy and practices” the influencing factors of teacher emotions could be explored at “international, national, federal and institutional levels as well as a range of relationships with key stakeholders such as school leaders, colleagues, students and parents and other community representatives” (Hofstadler et al., 2021, p. 2).

^3^However, including a few more reference variables (e.g., gender, highest educational qualification and other socio-biographical factors) together with the focal variables into regression analyses will facilitate a better understanding of the influence of focal variables on the dependent variable.

^4^Project 211, initiated by China’s Ministry of Education in 1995, provides special funds to support the building of 100+ tertiary institutions into top universities of an international standard. Project 985 is another government-initiated funding scheme initiated in 1998; as this scheme is more selective than Project 211, the universities listed in the former are fewer in number and hence more prestigious than those in the latter (Kong and Wei, 2019).

^5^This sample size was based on a rule of thumb that an exploratory survey-based study should have at least 100 participants (Wei, 2023).

^6^With high validity and reliability, Chen’s (2016, p. 74) scale represents “a new quantitative means to investigate teacher emotions in future research.” It turns out that some recent studies (e.g., Atmaca et al., 2020; Zhang and Tsang, 2021) have utilized this scale.

^7^EFA was used to explore the inherent structure of the revised TEI model with Principal Component Analysis as the factor extraction method in SPSS 25.0. The KMO test (0.842), Bartlett’s test of sphericity [χ^2^(300) = 1773.32, *p* < 0.001] indicated that the data could be used for factor analysis. During this procedure, two items (“I feel frustrated when my promotion is stuck by stiff policies.” and “I feel angry as I am forced to be involved in the teaching reform.”) were deleted in the model because the regression loading of the item was lower than 0.40. Five factors, accounting for 68.91% of the teacher emotion variance, were extracted.

^8^To measure effect sizes, *r* was calculated for the *t*-tests. All the effect sizes exceed the large/strong cut-offs (*r* = 0.14) according to the topic-specific effect size benchmark system proposed by Wei and Hu (2019). According to the system, the small/weak, typical (medium), large/strong, and very large/strong cut-offs for *r* are 0.07, 0.10, 0.14, and 0.30, respectively (Luo and Wei, 2021).
